# Prevalence and Determinants of Condom Non‐Use During Last Sexual Encounter Among Unmarried Women of Reproductive Age in Tanzania: Findings From a National Survey

**DOI:** 10.1002/puh2.70213

**Published:** 2026-04-06

**Authors:** Fabiola V. Moshi, Jovin R. Tibenderana, Gloria Kagaruki, Khadija Haddy, Jomo Gimonge, Sanun Ally

**Affiliations:** ^1^ Department of Clinical Nursing School of Nursing and Public Health University of Dodoma Dodoma Tanzania; ^2^ Department of Public Health St. Francis University College of Health and Allied Sciences Ifakara Morogoro Tanzania; ^3^ Africa Academy for Public Health Dar es Salaam Tanzania

**Keywords:** condom and Tanzania, condom non‐use, reproductive age, unmarried women

## Abstract

**Background:**

Condoms are central to preventing sexually transmitted infections (STIs) and unintended pregnancies. Despite awareness, use remains suboptimal among women in Tanzania. This study assessed prevalence and determinants of condom non‐use during the last sexual encounter among unmarried, sexually active women using nationally representative data.

**Methods:**

We conducted a cross‐sectional analytical study using data from the 2022 Tanzania Demographic and Health Survey (TDHS). A total of 2146 unmarried and sexually active women aged 15–49 years were included. The primary outcome was self‐reported condom non‐use during the last sexual encounter. Descriptive statistics and log‐binomial regression were used to identify factors associated with condom non‐use, adjusting for sociodemographic variables and survey weights.

**Results:**

The prevalence of condom non‐use during the last sexual encounter was 19.07%. Regional variation was noted, with the highest prevalence in the Lake zone (4.85%) and the lowest in Zanzibar (0.13%). After adjustment, secondary or higher education was associated with lower odds of condom non‐use (aRR = 0.61; 95% confidence interval [CI]: 0.38–0.97), as was having multiple sexual partners (aRR = 0.39; 95% CI: 0.20–0.76). Conversely, women with two or more children had increased odds of non‐use (aRR = 2.12; 95% CI: 1.26–3.56).

**Conclusion:**

Condom non‐use among unmarried women remains a significant public health concern in Tanzania. Education, parity, and sexual behavior patterns are key determinants. Interventions should prioritize comprehensive sexuality education, empower women in reproductive decision‐making, and address sociocultural barriers that hinder condom negotiation and use.

## Graphical abstract

Nearly one in five unmarried Tanzanian women did not use a condom during their last sexual encounter. Education, parity, and sexual behavior significantly influence non‐use, highlighting the need for targeted sexuality education and empowerment interventions to improve condom uptake and reduce STI and unintended pregnancy risks.

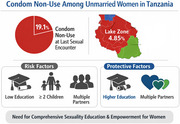

## 1 Background

Condoms play a vital role in public health strategies aimed at preventing or reducing sexually transmitted infections (STIs) and unintended pregnancies [1]. They are affordable, cost‐effective, and, when used correctly and consistently, offer high protection (80%–90%) against STIs, including HIV [1, 2]. Despite their high risk of STIs due to factors such as sexual activities, multiple sexual partners, and sociocultural and economic influences, consistent female condom use is not widely practiced among women of reproductive age [2, 3]. This underutilization poses a significant public health challenge, especially in resource‐limited settings like Tanzania.

Globally, the consistent use of condoms remains low, ranging between 4% and 52.4% among reproductive‐aged individuals [4]. Alarmingly, young adults are responsible for over 50% of new HIV transmissions, largely due to inconsistent condom use [4]. Each year, an estimated 357 million curable STIs occur among reproductive‐aged individuals, with 417 million women infected with herpes simplex virus type 2 and 291 million with human papillomavirus (HPV) [5].

In sub‐Saharan Africa, STIs, including HIV, remain leading causes of morbidity and mortality among young people, with unsafe sexual intercourse being the primary transmission route [6, 7, 8]. Tanzania mirrors this trend, facing a high burden of STIs and HIV among women [9], as the prevalence is reported to be around 4.7% and 5.6%, respectively [10, 11], yet the overall prevalence of condom non‐use remains poorly documented.

For women, STIs that are acquired as the results of condom non‐use may result into the health consequences, including infertility, chronic pelvic pain, and pregnancy complications, such as abortion, preterm labor, low birth weight, and stillbirth [12, 13]. Beyond health, STIs exert socioeconomic consequences by burdening healthcare systems and affecting women's overall quality of life [14].

In Tanzania, several studies have explored factors influencing condom use, identifying education level, age, substance use, and the nature of sexual partnerships as key determinants [15, 16]. Additionally, public health efforts have promoted condom distribution and awareness, especially among youth [3]. However, these initiatives often lack robust implementation and monitoring.

Despite ongoing interventions, a clear national picture of condom non‐use prevalence and associated factors among women of reproductive age is lacking. Most studies in Tanzania are limited in scope, with few using robust methodological designs or national datasets [16, 17]. These gaps hinder the formulation of evidence‐based policies and targeted interventions needed to reduce STI prevalence and improve condom use among Tanzanian women.

The main objective of this study is to determine prevalence and determinants of condom non‐use during last sexual encounter among unmarried women of reproductive age in Tanzania. Addressing condom non‐use among unmarried women is critical for achieving global and national targets such as Sustainable Development Goal 3.7 that aim to ensure universal access to sexual and reproductive healthcare services by 2030. The findings of this study will provide critical insight to policymakers and other stakeholders in designing an effective intervention aimed at reducing the incidence of STI and promoting condom use among women of reproductive age in Tanzania.

## 2 Materials and Methods

### 2.1 Study Setting and Period

The study utilized the 2022 Tanzania Demographic and Health Survey (TDHS) data, a national cross‐sectional survey carried out every 5 years [18]. Tanzania, the biggest country in East Africa, spans an area of 940,000 km^2^, including 60,000 km^2^ of inland water. According to the current census the estimated population of Tanzania stood at 61,741,120, with an annual population growth rate of 3.2% [19, 20]. Tanzania has diverse cultural and religious context regarding condom non‐use. Patriarchal norms exists in many communities but their influence varies by settings and young people's behavior reflects both traditional expectations and changing norms due to education and urbanization.

### 2.2 Study Design and Data Source

This study was an analytical cross‐sectional study. Data were sourced from the TDHS, which was funded by the US Agency for International Development, and implementation is carried out by the Ministry of Health (MoH) in Tanzania Mainland and Zanzibar, as well as the National Bureau of Statistics (NBS), the Office of the Chief Government Statistician (OCGS), with technical support provided by ICF International [19, 20, 21, 22].

### 2.3 Data Collection Procedure

The surveys used a standardized questionnaire, consistent across all countries, to collect data from women aged 15–49. The questionnaire is typically translated into the main local languages of the participating countries. As per DHS procedures, both the original English version and the translated versions are pretested in English and local dialects to ensure accuracy. During this pretesting stage, field staff participate in interactive discussions about the questions, offering suggestions to refine all language versions. After the practical fieldwork, a debriefing session is held with the pretest team, and the questionnaires are revised on the basis of the feedback and observations gathered during this process [18].

### 2.4 Sampling Procedure and Size

The survey used face‐to‐face interviews with structured questionnaires and adopted a stratified, multistage cluster sampling design to collect information on a range of topics, including population health, neonatal mortality, health behaviors, nutritional status, family planning, and demographic characteristics. In the initial stage, 629 clusters were identified, from which households were then selected. Among these clusters, 26 households were systematically chosen to be representative from each cluster, resulting in a total of 16,354 households included in the survey. Eligibility for inclusion was based on the presence of all women aged 15–49 years in the selected household on the night prior to the interview. Further details on the sampling procedure and design have been previously documented [18]. A total of 2146 unmarried and sexually active women (*defined as women who reported having had sexual intercourse within a month prior the survey*) who had information on all the variables of interest were included in the study.

The DHS data are freely available for download at https://dhsprogram.com/data/available‐datasets.cfm.

### 2.5 Study Variables

The outcome variable of this study was condom non‐use. This was from the question “was condom used during last sex with most recent partner?”. Response is coded as 0 = “Yes” and 1 = “No”. The explanatory variables were age of the woman, education, occupation, residence, wealth index, head of household age, media exposure. Age was recorded as 15–24, 25–34, and 35–49. Wealth status was categorized as poor, middle, and rich. Education was classified into three categories: no education, primary education, secondary education, or higher education. Employment was categorized in working and not working. Residence was categorized into urban and rural. Age of head of household was categorized into 15–24, 25–34, and 35–49. Visited health facility in the last 12 months was categorized into “Yes” and “No”. The number of sexual partners was categorized into no sexual partner, one or more than one. A new variable of media exposure, generated from household, has either TV or radio. Our generated study variables and coding were based on previous literature [15, 23, 24].

## 3 Statistical Analysis

We used Stata software, version 18.0 (Stata Corporation, College Station, TX, USA) for all statistical analyses. Initial presentation of condom non‐use was done using percentages to show its proportion among unmarried women. Before conducting the regression analysis, we assessed potential collinearity among the variables, using the variance inflation factor (VIF) and results suggested no significant multicollinearity concerns. To investigate the determinants of condom non‐use, we employed log‐linear regression (generalized linear model with the log‐binomial family and log‐link function) that was used because the outcome of interest (non‐condom use) was common >10% and hence using logistic regression would overestimate of effect sizes. Additionally, the modified poison model failed to converge. Crude and adjusted odds ratios (AOR) with corresponding 95% confidence intervals (CIs) were fit for each model. A significance level of *p* < 0.05 was used for all statistical tests. Furthermore, we weighed all analyses to account for disproportionate sampling and non‐response.

### 3.1 Ethical Clearance

This study used secondary data from the TDHS. The ethical approval for the original survey was obtained from the National Institute for Medical Research (NIMR) and the ICF International Institutional Review Board. Written informed consent was obtained from all participants prior to data collection, with parental or guardian consent for respondents aged 15–17 years. The survey ensured privacy, confidentiality, and anonymization of participant information. Permission to access and use the TDHS dataset for this secondary analysis was granted by the DHS Program. Further detailed explanation regarding the methodology and ethical considerations of the DHS program can be found in the published reports of the TDHS [18, 22].

## 4 Results

Table 1 describes the study participants with median age (IQR) 17 (16–19) years, almost all (97.2%) were aged 15–24 years. The majority (64.8%) had secondary or higher education. Slightly less than three quarter (69.5%) were not working. More than three‐fifth (60.6%) were living in rural. More than half (54.7%) of the study participants were from rich household.

**TABLE 1:** Characteristics of the study participants (*N* = 2146) (weighted).

Variable	Frequency (*n*)	Percentage
**Age in years**		
Median (IQR)	17 (16–19)	
15–24	2085	97.2
25–49	61	2.8
**Education**		
No education	87	4.0
Primary	668	31.2
Secondary/higher	1391	64.8
**Employment**		
Not working	1492	69.5
Working	654	30.5
**Residence**		
Urban	846	39.4
Rural	1300	60.6
**Wealth index**		
Poor	557	25.9
Middle	415	19.3
Rich	1174	54.7
**Media exposure**		
No	858	40.0
Yes	1288	60.0
**Sex of household head**		
Male	1521	70.9
Female	625	29.1
**Visited health facility last 12 months**		
No	1622	75.6
Yes	524	24.4
**Parity**		
1	2044	95.2
2+	102	4.8
**Age of head of household**		
15–24	24	1.1
25–34	135	6.3
35–49	1987	92.6
**Parity**		
1	2111	98.4
2+	35	1.6
**Number of sexual partners**		
No sexual partner	39	1.8
1	118	5.5
>1	1989	92.7

### 4.1 Prevalence of Condom Non‐Use Use Among Unmarried Sexually Active WRA

**FIGURE 1:**
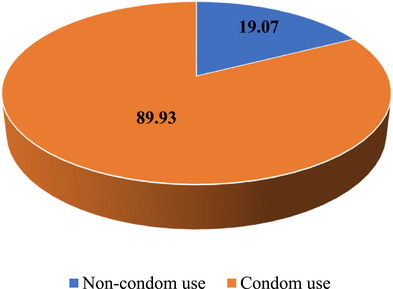
Prevalence of condom non‐use among unmarried women of reproductive age.

Among unmarried, sexually active women in Tanzania, condom non‐use during the last sexual encounter with their most recent partner was reported to be 19.07% (Figure 1).

### 4.2 Prevalence of Condom Non‐Use Among Unmarried Women by Zones

**FIGURE 2:**
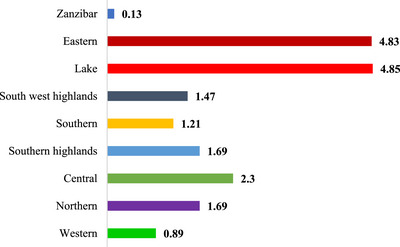
Prevalence of condom non‐use among unmarried by zones.

The highest prevalence of condom non‐use was observed in Lake zone (4.85%) and the lowest was observed in Zanzibar (0.13%) (Figure 2).

### 4.3 Determinants of Condom Non‐Use Among Unmarried Women in Tanzania

After adjusting for other factors, three determinants were significantly associated with condom non‐use during the last sexual encounter among unmarried women. Women with secondary or higher education had 39% less odds of condom non‐use (OR = 0.61, 95% CI: 0.38–0.97) compared to those who had no education. Women who had multiple sexual partners had 61% less odds of condom non‐use compared to those who had none (OR = 0.39, 95% CI: 0.20–0.76). Lastly, women who had given birth more than once were more likely to report condom non‐use (OR = 2.12, 95% CI: 1.26, 3.56) compared to their counterparts (Table 2).

**TABLE 2:** Determinants of condom non‐use among unmarried women in Tanzania (*N* = 2146).

Variable	Crude RR (95% CI)	Adjusted RR (95% CI)
**Age categories**		
15–24	Ref	Ref
25–49	1.36 (0.89–1.59	1.203 (0.91–1.42)
**Residence**		
Urban	Ref	Ref
Rural	1.30 (1.03–1.64)	0.96 (0.70–1.31)
**Educational level**		
No education	Ref	Ref
Primary	0.54 (0.35–0.82)	**0.62 (0.40–0.94**)
Secondary or higher	0.44 (0.29–0.68)	**0.61 (0.38–0.97)**
**Wealth index**		
Poor	Ref	Ref
Middle	0.72 (0.51–1.01)	0.79 (0.55–1.13)
Rich	0.57 (0.43–0.75)	0.73 (0.47–1.11)
**Employment status**		
Not working	Ref	Ref
Working	0.98 (0.75–1.28)	0.97 (0.74–1.28)
**Number of sexual partners**		
No	Ref	Ref
1	0.38 (0.21–0.70)	**0.46 (0.25–0.84)**
>1	0.30 (0.15–0.59)	**0.39 (0.20–0.76)**
**Media exposure**		
No	Ref	Ref
Yes	0.72 (0.57–0.91)	0.89 (0.67–1.17)
**Parity**		
1	Ref	Ref
+2	2.7 (1.6–4.5)	**2.12 (1.26–3.56)**

*Note:* The bold elements signifies statistically significant.

## 5 Discussion

The study aimed to assess prevalence and determinants of condom non‐use during last sexual encounter among unmarried women of reproductive age in Tanzania. Prevalence was found to be 19.07%. Determinants that significantly associated with condom non‐use among unmarried women of reproductive age in Tanzania are education, number of sexual partners, and parity.

There are a number of interconnected reasons behind the observed prevalence of unmarried women not using condoms. First, some women might feel that their relationships are stable or trustworthy, which would make them less inclined to use condoms [25]. Second, women may neglect the value of condoms for STI and HIV protection if they rely too much on alternative forms of contraception, like injectables or implants [26]. Third, women's ability to negotiate condom use is limited by deeply ingrained cultural and gender conventions that frequently give male partners the last say in decision‐making [27], particularly in situations when asking for condom use could be seen as an indication of infidelity or mistrust. Another explanation could be perceived reduced pleasure and not being able to enjoy sex with condom [28]. It should however be clear that the variable measures condom non‐use at last sex, and not their overall behavior regarding condom use, a woman may be a consistent condom user but happened not to use it during her last encounter which may be intentional or non‐intentional.

Every educated woman is the result of making well‐informed decisions, particularly in regards to safeguarding her health [29]. How unmarried women manage their sexual and reproductive life is significantly shaped by education, especially at the secondary level and beyond. Educated women are better able to avoid undesirable outcomes such as STIs and unexpected pregnancies because they have more access to health information [30], more decision‐making power, and more confidence when negotiating safe sex. Beyond merely imparting academic knowledge, education fosters critical thinking, self‐reliance, and access to chances for personal growth [31], all of which together lower the risk of having unsafe sex. These observations emphasize education as a strategic public health intervention with wide‐ranging advantages, rather than just as a social good. These findings align with previously published evidence [32].

The more partners a woman has, the more vigilant she becomes about sexual protection. In Tanzania, unmarried women who reported having one or more sexual partners had a considerably lower likelihood of having unprotected sex than those who did not. This tendency might be the result of heightened risk perception; women who have had sex are probably more conscious of the possible risks, such as HIV, STIs, and unwanted pregnancies, and are therefore more likely to use condoms. Remarkably, the protective effect was even more pronounced for those who had several sexual partners, indicating that more sexual exposure can potentially encourage more watchful behavior rather than riskier activity. Different findings have been published elsewhere [16, 17]; the difference can be attributed to the smaller sample size in the previous study, but also the current study is nationally representative.

Contraceptive decision‐making among women with multiple births is influenced by a constellation of factors that go beyond individual preference, reflecting deeper social, relational, and structural dynamics. The observed lower condom use in this group may be rooted in a false sense of security within long‐term relationships, where trust and perceived monogamy often reduce the perceived need for barrier protection. Additionally, many multiparous women turn to long‐acting or hormonal contraceptives for pregnancy prevention, which, while effective, offer no protection against STIs [33]. Cultural expectations and gender power imbalances further complicate the ability of these women to negotiate condom use, particularly in male‐dominated settings where reproductive decisions are heavily influenced by partners.

### 5.1 Strength and Limitations

We used data from the most recent DHS, which is well‐known for its reliable procedures and validation in a large number of research. Our findings are generally applicable to Tanzanian unmarried women from other African nations because we used nationally representative data. The use of secondary data limited our analysis to the variables present in the datasets, excluding important elements such as patriarchal standards and cultural influences that may influence women's STI risks. This study is not without limitations though, this study composed of predominant sample of unmarried women aged 15–24 years, consequently the findings are most applicable to younger women and caution is warranted while interpreting the results. Additionally, using secondary data limited our research to the variables present in the datasets, leaving out important elements that could influence women's condom non‐use. Furthermore, causal interpretations are not possible because to the DHS's cross‐sectional nature.

## 6 Conclusion and Recommendations

This study shows that condom non‐use among unmarried women in Tanzania is largely driven by societal and structural factors such as gender norms, perceived relationship security, and reliance on non‐barrier contraception; therefore, sexual and reproductive health programs should implement gender‐transformative interventions that address harmful norms and misconceptions around condom use. Education was found to enhance women's autonomy and informed sexual health decision‐making, underscoring the need to strengthen comprehensive sexuality education and life‐skills programs that emphasize condom negotiation and risk perception. Persistent power imbalances within sexual relationships continue to limit women's ability to negotiate condom use, highlighting the importance of actively engaging men and couples to promote equitable and shared decision‐making. Finally, as condom availability alone is insufficient to ensure use, a multisectoral approach that integrates condom access with women's empowerment and community norm–change strategies is essential.

## Author Contributions

**Fabiola V. Moshi**: conceptualization, methodology, writing – original draft, writing – review and editing. **Jovin R. Tibenderana**: formal analysis, methodology, writing – original draft, writing – review and editing. **Sanun Ally Kessy**: formal analysis, methodology, writing – original draft, writing – review and editing. **Jomo Gimonge**: writing – original draft, writing – review and editing. **Gloria Kagaruki**: writing – original draft, writing – review and editing. **Khadija Haddy**: writing – original draft, writing – review and editing.

## Funding

The authors have nothing to report.

## Ethics Statement

Permission to use the data was obtained from the DHS Program/ICF International via http://www.dhsprogram.com.

## Conflicts of Interest

The authors declare no conflicts of interest.

## Data Availability

The datasets generated and/or analyzed during this study are available from the corresponding author upon reasonable request.
